# ZooArchNet: Connecting zooarchaeological specimens to the biodiversity and archaeology data networks

**DOI:** 10.1371/journal.pone.0215369

**Published:** 2019-04-12

**Authors:** Michelle J. LeFebvre, Laura Brenskelle, John Wieczorek, Sarah Whitcher Kansa, Eric C. Kansa, Neill J. Wallis, Jessica N. King, Kitty F. Emery, Robert Guralnick

**Affiliations:** 1 Florida Museum of Natural History, University of Florida, Gainesville, Florida, United States of America; 2 Museum of Vertebrate Zoology, University of California, Berkeley, California, United States of America; 3 Open Context, San Francisco, California, United States of America; 4 Archaeological Research Facility, University of California, Berkeley, California, United States of America; Institut Català de Paleoecologia Humana i Evolució Social (IPHES), SPAIN

## Abstract

Interdisciplinary collaborations and data sharing are essential to addressing the long history of human-environmental interactions underlying the modern biodiversity crisis. Such collaborations are increasingly facilitated by, and dependent upon, sharing open access data from a variety of disciplinary communities and data sources, including those within biology, paleontology, and archaeology. Significant advances in biodiversity open data sharing have focused on neontological and paleontological specimen records, making available over a billion records through the Global Biodiversity Information Facility. But to date, less effort has been placed on the integration of important archaeological sources of biodiversity, such as zooarchaeological specimens. Zooarchaeological specimens are rich with both biological and cultural heritage data documenting nearly all phases of human interaction with animals and the surrounding environment through time, filling a critical gap between paleontological and neontological sources of data within biodiversity networks. Here we describe technical advances for mobilizing zooarchaeological specimen-specific biological and cultural data. In particular, we demonstrate adaptations in the workflow used by biodiversity publisher VertNet to mobilize Darwin Core formatted zooarchaeological data to the GBIF network. We also show how a linked open data approach can be used to connect existing biodiversity publishing mechanisms with archaeoinformatics publishing mechanisms through collaboration with the Open Context platform. Examples of ZooArchNet published datasets are used to show the efficacy of creating this critically needed bridge between biological and archaeological sources of open access data. These technical advances and efforts to support data publication are placed in the larger context of ZooarchNet, a new project meant to build community around new approaches to interconnect zoorchaeological data and knowledge across disciplines.

## Introduction

Interdisciplinary collaborations hold the key to addressing the complex human-environmental relationship and its influence on biodiversity at broad spatial, temporal, and cultural scales [1–6]. Catalytic in supporting such collaborations has been recent growth in open sharing of biodiversity data, including that from modern (neontological) through deep time (paleontological) specimens, as well as the networks and tools for accessing and interpreting these data. Key efforts to mobilize biodiversity data have made available over a billion specimen records in a global network of data publishing/access platforms linked through the Global Biodiversity Information Facility (GBIF; gbif.org). Equally important has been the community development of standards (e.g., Darwin Core [7]) and integration tools (e.g., IPT [8]) to encourage compilations of biodiversity knowledge (e.g., the Map of Life [9]) for global research efforts. The biodiversity data network provides rich digitally-accessible content, typically over broad spatial extents, and increasingly, over broad time scales, that can feed into modeling frameworks capable of documenting human interactions with the environment from the earliest periods of our history [10,11].

Similarly, recent initiatives in archaeological data sharing technologies have made available enormous libraries of openly-accessible archaeological data documenting the long and culturally diverse global history of human-environmental relationships. The breadth of data and content includes digital documents, images, and data in original source formats (*e*.*g*., Archaeological Data Service (ADS) (archaeologydataservice.ac.uk), Data Archiving and Networked Services (DANS) (dans.knaw.nl/en), The Digital Archaeological Record (tDAR) (tdar.org), The Strategic Environmental Archaeology Database (SEAD) (snd.gu.se/en/catalogue/study/EXT0021)), as well as combined datasets with highly standardized ontologies (e.g., Canadian Archaeological Radiocarbon Database (CARD) (canadianarchaeology.ca), Digital Archaeological Archive of Comparative Slavery (DAACS) (daacs.org), Digital Index of North American Archaeology (DINNA) (ux.opencontext.org/archaeology-site-data/), North Atlantic Biocultural Organization (NABO) (nabohome.org)), and in data publication and research portals that both archive data and also link among repositories (e.g., Ariadne (ariadne-eu.org), Open Context (opencontext.org)). Innovations in archaeoinformatics (and data research in other social science and humanities disciplines) have emphasized Linked Open Data (LOD) technologies which allow annotation with persistent identifiers that connect data, ontologies, and resources in lieu of strict conformance to predetermined semantic models or standards. Instead of distributed data networks, these technologies emphasize automated entity reconciliation to develop and maintain cross references among records in multiple sources. These technologies facilitate highly granular networking among cultural heritage information systems from around the world despite differences in research approaches, language, data models, and software.

Despite this progress across disciplines, less emphasis has been explicitly placed on the integrated assembly of biodiversity and cultural heritage information, especially for zooarchaeological specimens. In this paper we detail our recent interdisciplinary efforts to integrate zooarchaeological specimens, with their highly intertwined biological/cultural information, into existing biodiversity networks while also maintaining persistent linkages to archaeoinformatics platforms. We dub this collaborative work ZooArchNet. Zooarchaeological (or archaeofaunal) specimens are the remains of animals, including vertebrate and invertebrate taxa, recovered from, or in association with, archaeological contexts of deposition or surrounding landscapes. These are inherently both biological specimens and cultural artifacts, making them a significant source of data that are representative of the long history of human interaction with the environment. The physical scope of zooarchaeological specimens is diverse and includes macro- and micro-zooarchaeological specimens composed of archaeologically preserved bone, shell, exoskeletons, teeth, hair or fur, scales, horns or antlers. Objects created from animal remains, such as bone pins, shell beads, and preserved animal hides, and geochemical (e.g., isotopes) and biochemical (e.g., aDNA) signatures derived from faunal remains, are also included in zooarchaeological research. These types of remains are records of ancient biodiversity, or the presence, availability, and selection of represented animals during the period of occupation of the archaeological site from which they are recovered. But they are also records of the culturally mediated human perceptions, decisions, and practices guiding animal exploitation, manipulation, consumption, translocation, and use across variable environmental settings and climatic conditions.

The combination of biological and cultural data over time is vital for reconstructing shifting biodiversity baselines from the earliest periods of human occupation to accurately document the scale and rate of human impact. This combined data is also a vital source of information on human responses to biodiversity (or resource) loss, which is essential to fully understand how human-environment coupled systems work [12–14]. Zooarchaeological data provide long-term perspectives on many critical issues underlying the current biodiversity crisis, including overexploitation, animal manipulation, landscape modification, species extirpation and extinction, and human response to environmental change [15–21]. Biological and cultural data represented by archaeofaunal specimens are not mutually exclusive, and taken together they provide unique opportunities to challenge and push conceptual boundaries and research parameters in biodiversity research from both biological and anthropological perspectives (e.g., [22–26]). Nevertheless, the great research potential of zooarchaeological or other environmental archaeology data within biodiversity initiatives is yet to be fully realized due to a lack of cross-disciplinary data-sharing infrastructure between biological and archaeological informatic communities.

Given the unique nature of zooarchaeological specimens, a key question we address in this work is how to build networks that can more effortlessly exchange cultural and biodiversity content in ways that best support collaboration across disciplines. Doing so is challenging, requiring a willingness to negotiate variable ontological and data traditions of how to characterize and present environmental archaeological records, including zooarchaeological specimen data, as biodiversity data—particularly across multiple disciplines spanning life and social science perspectives. Here, we show how such data networks can form via collaborations that emphasize linked open data frameworks. ZooArchNet leverages biodiversity informatics infrastructure created by the biodiversity data publisher VertNet (vertnet.org) [27], biological data reporting standards (Darwin Core [7]), and various biodiversity data management tools. Acting as a bridge, ZooArchNet embraces specimen-specific biological and cultural data, such as context and chronology, through innovations in biodiversity data mobilization workflow and publication practices. Vitally, it also draws on developments in the use of some linked open data practices (which favor the use of highly granular persistent links among entities), such as open data and persistent identifier, to create strong links with an exemplar archaeoinformatics platform, Open Context, and to reporting standards shared by both biodiversity and archaeology informatics platforms (e.g., Uberon [28]). The ZooArchNet framework is, by design, extensible to other biodiversity publishing platforms, including those already integrated with VertNet (e.g., iDigBio (idigbio.org)), and others that are not (e.g., The Paleobiology Database (paleobiodb.org), Neotoma (neotomadb.org)), as well as to other archaeological data repositories and publishers (e.g. DINAA, DAACS, tDAR). ZooArchNet thus critically accommodates the integration of zooarchaeological records to the biodiversity record with their associated biological and cultural information intact and begins to bridge existing gaps between the life and social sciences and their disciplinary communities.

Our focus in this paper is on the mechanics of mobilizing zooarchaeological data utilizing existing biodiversity data publishing tools and linked open data innovations. The development of this process represents a multi-year effort of the ZooArchNet collaborators to align practices to properly represent data for both biodiversity and archaeological communities. Our discussion emphasizes the importance of data standards in facilitating interoperability between different data sharing networks, and the value of shared identifiers as a key means to link across those standards, as well as among datasets and archives from different disciplines. We close by discussing how ZooArchNet, with its primary focus on zooarchaeological specimens and their associated data, helps facilitate innovations needed in both the biodiversity informatics and archaeoinformatics communities.

## Methods and results

### Overview

**Fig 1:**
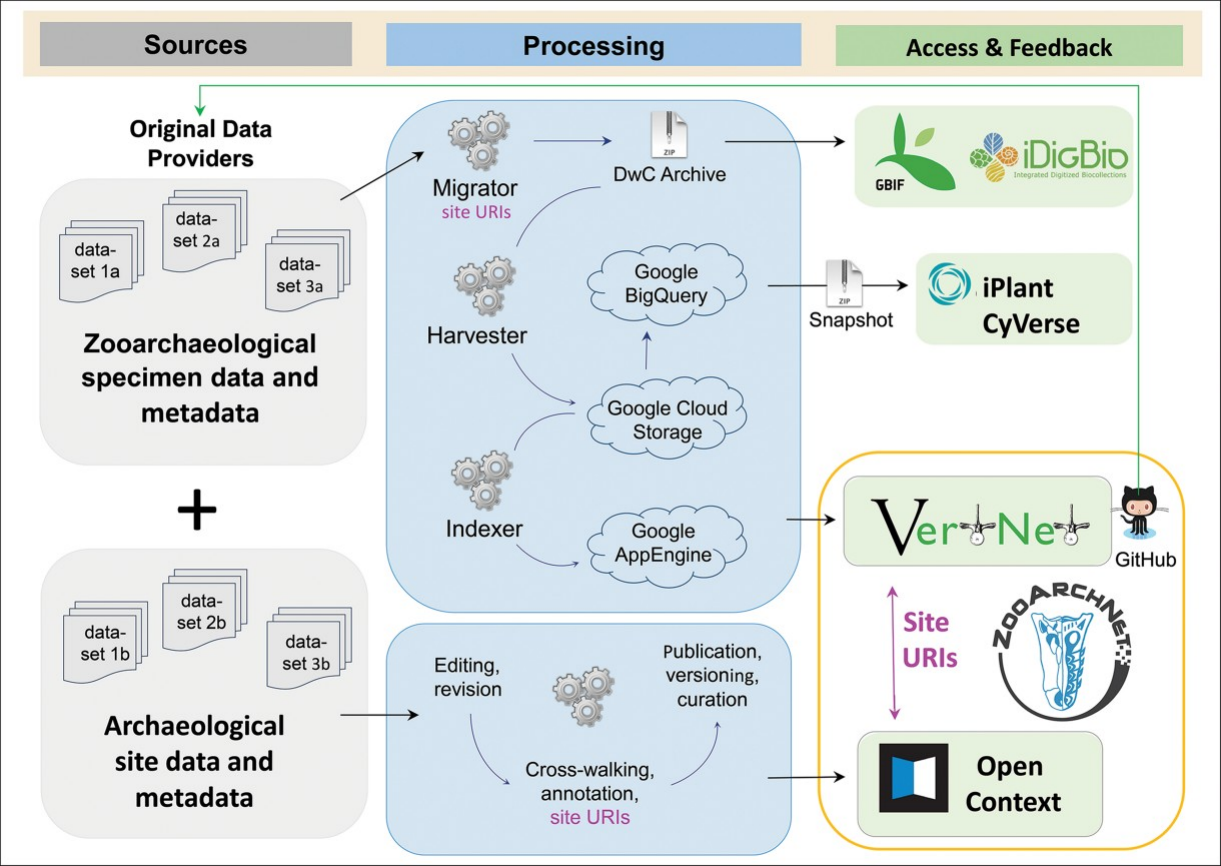
ZooArchNet workflow.

ZooArchNet utilizes existing workflows developed in the biodiversity informatics and archaeoinformatics communities, but critically provides a means to link between them, summarized in Fig 1. Zooarchaeological specimen data are mobilized and published using tools in biodiversity informatics, but adapted and extended, as depicted in the top panel of the figure. An independent step is to publish site information and provide published specimen data utilizing archaeoinformatics repositories, in this case exemplified by Open Context (bottom panel, Fig 1). Currently, ZooArchNet specimen records are published through VertNet [27] and its existing portal, as discussed more below. Zooarchaeological specimen records are a subset of the larger biological specimen database available through VertNet and connected portals (e.g., GBIF). ZooArchNet and VertNet work synergistically; ZooArchNet provides a needed framework for the organization and presentation of explicitly zooarchaeological data and VertNet provides structure for the publication and discovery of the records in a biodiversity format. Efforts are underway to create a ZooArchNet portal (see zooarchnet.org) in the future to serve as a more direct point of data contribution and discovery, with more tailored searching mechanisms for archaeological relevant content. However, the published data records themselves will not differ. The specifics we present here for publishing zooarchaeological specimen data are best practices developed during work with multiple exemplar datasets curated in the Florida and Environmental Archaeology Divisions of the Florida Museum of Natural History, and now published as part of ZooArchNet via VertNet (Table 1).

Zooarchaeological specimen data will be mobilized in biodiversity data networks (exemplified by GBIF, iDigBio, VertNet, and the proposed ZooArchNet) using existing VertNet technologies. When possible, specimen data will be linked to archaeological site data, and thus to the broader framework of Linked Open Data (not shown), through site URIs minted in the archaeological data publishing workflow as exemplified here by Open Context.

**Table 1:** Exemplar ZooArchNet datasets published in VertNet with links to Open Context.

Archaeological collection metadata^a^	Description of archaeological site linked to the archaeological collection metadata
FM Curatorial Range: Florida Archaeology; Collection Accession #: 2012–018; Archaeological Site #: 8CO326; Archaeological Site Name: Parnell Mound	Vertebrate and invertebrate remains recovered from a Suwannee Valley archaeological culture site near White Springs, Florida. The collection was excavated in 2012 from a single pit feature approximately 2.5m by 3m wide representing a single depositional event. The zooarchaeological specimen records are associated with an AMS assay on charred wood from the feature that yielded a radiocarbon age of 850+/- 30 years BP, which gives a 2-sigma calibrated date range of AD 1050 to 1080 (5.2%) and 1150 to 1260 (90.2%) (using calibration curve IntCal13 [29]).
FM Curatorial Range: Environmental Archaeology; Collection Accession #: 221; Archaeological Site #: 8SU65; Archaeological Site Name: Baptizing Springs	Vertebrate and invertebrate remains recovered from a Suwannee Valley archaeological culture site near White Springs, Florida. The collection was excavated in 2012 from a single pit feature approximately 2.5m by 3m wide representing a single depositional event. The zooarchaeological specimen records are associated with an AMS assay on charred wood from the feature that yielded a radiocarbon age of 850+/- 30 years BP, which gives a 2-sigma calibrated date range of AD 1050 to 1080 (5.2%) and 1150 to 1260 (90.2%) (using calibration curve IntCal13 [29]).
FM Curatorial Range: Environmental Archaeology; Collection Accession #: 19; Archaeological Site #: 8VO24; Archaeological Site Name: Tick Island	Vertebrate and invertebrate remains recovered from multiple contexts associated with site occupation spanning from approximately 4,000 BC to 1 AD. Excavated during the 1970's, the Tick Island site is located in Volusia County, Florida, near the St. Johns River.
FM Curatorial Range: Environmental Archaeology; Collection Accession #: 067; Archaeological Site #: 8FL216; Archaeological Site Name: North Midden	Vertebrate and invertebrate remains from a late Mount Taylor period archaeological site located along the Intracoastal Waterway in Flagler County, Florida. The site was excavated in 2006 and the zooarchaeological dataset is a sample from the bottom layers of a pit feature. The zooarchaeological specimens are associated with a 2-sigma calibrated date range of 4970–4560 BP.

^a^All zooarchaeological data is from archaeological collections curated at the Florida Museum of Natural History (FM).

A key decision that guided our efforts was that zooarchaeological specimen data should be aligned as closely as possible to the Darwin Core standard [7], but should also be published with additional specimen-specific descriptive content critically needed for interpretation, including: element descriptions, chronology, material condition, source and location (i.e., provenience), as well as verbatim identifications that do not align directly with taxonomic or other categories (e.g., domestic animal breed categories, culturally specific body portions such as “haunch”, etc.). This creates standardized content for those parts of zooarchaeological specimen data that generally overlap with neontological or paleontological records, while also ensuring that key archaeological contextual information is available at the point of data publication. It is important to note that aggregators such as VertNet and GBIF were already publishing zooarchaeological specimen records (though not full data sets) prior to the ZooArchNet project (seehttp://portal.vertnet.org/o/mcz/ich?id=mcz-ich-39666 as an example). However, key archaeological data (e.g., archaeological context, chronology, skeletal element) are generally not associated with zooarchaeological specimens in these datasets resulting in what are essentially incomplete data records. The lack of such information significantly limits the utility of specimen records and masks the full biological and anthropological potential of the data (e.g., that an occurrence was in-situ or local to an archaeological site rather than being translocated and introduced to the location).

A second key decision to support a more robust collaborative use of the published zooarchaeological data was to provide additional archaeological data that, by association, gives interpretive value (e.g., associated artifact materials that identify the context of recovery as a locus of cooking, storage, or discard) through permanent and discoverable links to archaeological repositories and publishers such as Open Context, tDAR, Neotoma, or DAACS. These archaeoinformatics initiatives are steadily growing and in many cases already provide the essential associative information to better contextualize the published zooarchaeological specimen data. In cases where such archaeological information is not yet digitally available, the publication process can be simultaneous through VertNet/ZooArchNet and collaborating archaeological data publisher portals.

#### The VertNet publishing process

ZooArchNet specimen data are published using workflows developed for VertNet, detailed in Constable et al. [27] and Cicero et al. [30]. Since this process is critical for understanding choices made regarding publishing of zooarchaeology specimen data, we summarize certain aspects of it here. The first step in the VertNet data publishing process is determining what constitutes a “dataset” for publication. Biodiversity specimen data is published as specimen records (also called ‘occurrences’, all the information about a single organism or unit of organisms such as a “lot” of taxonomically identical specimens) organized by fields (categories of information such as age or collection locality). This is often a whole database of data about specimens within a collection or subcollection—for example in a neontological collection, data about all fish specimens curated in a larger vertebrate zoology collection (e.g., the Field Museum of Natural History Fish Collection—gbifdatasetid:afc30a94-6107-488a-b9c0-ba9c4fa68b7c). However, this choice can vary and be broader (including all specimens curated in a single institution such as the complete collections of the Australian Museum—gbifdatasetid:dce8feb0-6c89-11de-8225-b8a03c50a862), or be more granular, including only certain specimen forms (e.g., Field Museum of Natural History Bird Egg Collection—gbifdatasetid:43e0bba3-9edb-4b00-b490-84924e55a222), or specimens from a certain named collection (e.g., the Hildebrand collection of the Berkeley Museum of Vertebrate Zoology—gbifdatasetid:423d9318-4dd4-4d31-81cb-27778c44a3bc). Each dataset is provided with a persistent identifier.

The next step is standardizing the metadata about the dataset to be published, and aligning the dataset fields and their contents to the Darwin Core data standard which is used by all biodiversity data publishers and portals [7]. VertNet uses a profile schema based on the Ecological Metadata Language [31] to standardize dataset metadata. This includes fields to capture information about the datasets including the associated projects and people, the taxonomic, temporal, and spatial coverage of the specimens within the dataset, the general methods of data collection for the dataset, intellectual property rights for data sharing, etc. In the next VertNet workflow step, specimen record fields within the dataset are cross-walked to, or aligned with, the Darwin Core standardized vocabulary fields to capture specimen record level data describing such things as the collecting event during which the specimen(s) that compose the specimen record were acquired, the location where the specimen(s) were collected (including georeferenced locality and, for paleontological specimens, sometimes also geologic associations), information about the organism(s) (such as taxonomy, sex, life stage, specimen preparation type), and identifying catalog numbers by which the record is recognized by analysts/collection curators. Each occurrence is also provided with a unique identifier.

While Darwin Core fields capture content that is consistent across different types of natural history collections, there is often content specific to disciplines, or other assertions about specimens, that cannot be accommodated within the confines of the standard field structure. Darwin Core does provide property and notes fields that often are used to report that content. A key example is the Darwin Core term ‘dynamicProperties’, whose definition is “A list of additional measurements, facts, characteristics, or assertions about the record. Meant to provide a mechanism for structured content.” These fields will be discussed in more detail below.

Development of dataset-level metadata and cross-walking data fields to Darwin Core is often accomplished using a tool called the Integrated Publishing Toolkit or IPT [32], a Java-based application that provides computer-aided data publishing support. The IPT not only provides needed tools for creating datasets that conform to standards, but also accomplishes the next step which is to output Darwin Core Archives and landing pages describing those archives. These Darwin Core Archive outputs are self-extracting files containing Darwin Core formatted data and metadata. Data providers including researchers and data curators or their designated representatives “publish” these archives via the IPT, which announces their open availability and updates to subscribers via RSS feeds for each dataset. Automated processes “harvest” new or updated datasets and initiate indexing routines from VertNet to make these data available both in the VertNet portal itself (and eventually in the ZooArchNet portal), and also in networked portals such as iDigBio and GBIF (Fig 1). Though GBIF, VertNet, and iDigBio all use the same Darwin Core Archives to integrate and publish data through their portals, each portal can have specific indexing steps to help users better discover data in different contexts. For example, VertNet has custom tools to help index whether a specimen is a fossil, has associated media, has geographic coordinates for mapping, and, critical for the purposes here, whether the record is zooarchaeological in nature. VertNet also produces archives of all data from a particular taxonomic class and makes those accessible as snapshots for research through large-scale data repositories (CyVerse, cyverse.org; DataONE, dataone.org).

Data publishing is often a collaborative endeavor. The collaboration is typically between the data providers “publishers” (researchers and curators of specimen collections and their associated data), and the data mobilizers who develop and maintain the informatics infrastructure. Data mobilizers help data publisher make decisions, and guide them through the publishing process. The process, at least for datasets published through VertNet, is not linear and involves needed feedbacks. The VertNet team calls this process “migration”. Data migration involves checking draft versions of datasets to determine if content being mobilized is designated consistently to the standardized dwc fields, and, for some categories of information, that the internal content is standardized (e.g., taxonomies, locality), thus enhancing discovery. Though IPT publishing assures matching field names across datasets, that process does not standardize the content of most fields. As an example, the Darwin Core field “sex” is defined as “The sex of the biological individual(s) represented in the Occurrence” with a recommended best practice to use a controlled vocabulary e.g., “male”, “female”, “hermaphrodite”. However, values are often highly variable; a summary of existing records in VertNet found hundreds of variant ways that information about sex is represented (see https://soyouthinkyoucandigitize.wordpress.com/2013/07/18/data-diversity-of-the-week-sex/). Data migration is a means to help overcome this heterogeneity and involves looking up values of fields against a known list of possible variants and providing a canonical term as an alternate. This process never changes source data, but a report is presented to data publishers about how content could be standardized when published and any eventual changes are made by mutual agreement. For zooarchaeological specimens, the ZooArchNet collaborators have developed a special set of migration steps, as discussed below.

#### The ZooArchNet publishing process for zooarchaeological specimen data

A key starting point in publishing zooarchaeological datasets is to recognize that processes of zooarchaeological record digitization, organization, and presentation are variable within and between repositories and individual data providers. Zooarchaeological specimen data are generated and curated across a diversity of repositories and individuals, including museums, academic departments, commercial companies, state and federal agencies, as well as individual scholars and avocational practitioners. Zooarchaeological data are often curated by the analyst, sometimes the excavator who originally recovered the specimens, and sometimes only in a curatorial repository unassociated with either excavator or analyst. Moreover, there is considerable variability in how integrated zooarchaeological records and data are with associated archaeological finds, site chronology, and related metadata (e.g., [33]), all of which is essential to the effective interpretation of the zooarchaeological data for either biological or anthropological research. There is also great variability in how zooarchaeological specimens are identified and documented, including different types of physical records (e.g., hard copy paper files, electronic spreadsheets, photographs), organizational formats and databases, analytical observations/units recorded, and quality assessment protocols followed [34,35,36].

Zooarchaeological data reporting and digitization through ZooArchNet thus involves multiple steps and procedures related to data access, organization, and archaeological contextualization (e.g., [33,37]). Broadly, a goal of data digitization and organization efforts is to efficiently and effectively present all direct data from the zooarchaeological collection (e.g., collector name, date of collection, etc.), organism (e.g., taxon, age, sex, etc.), and specimen (e.g., element, element portion, side, etc.) within the context of archaeological provenience records (e.g., location of recovery, context and chronology of recovery, etc.) and methods of analysis (e.g., excavation strategy, sampling method, steps in specimen identification, etc.). These data are particularly important when reporting the analytical unit(s), geographic, temporal, and contextual scope of what constitutes a dataset and additional metadata.

#### Zooarchaeology, sites, and dataset designation

For zooarchaeological data mobilization, the first step is the same as for any other dataset–deciding what constitutes the “dataset” (or collection, record set, etc.). However, zooarchaeological data differs in one respect from other biodiversity data that is key to the first step of data designation and that is in the use of “site” as a culturally significant element with broad disciplinary recognition. The archaeological definition of specific horizontal and vertical spatial location data and its interpretation as cultural “sites” and/or “strata” are not part of the typical neontological dataset, although they are somewhat consistent with paleontological time-space location information. Neontological specimens are typically conceptualized in terms of their collection location as defined by modern locality terminology (e.g., W River near intersection of X and Y roads in Z county) and are considered representative of the moment at which they are collected (e.g., collection date). Although the definition of geographic locality and date of collection is also important for zooarchaeological records, equally relevant are the cultural designation of the ancient residential unit and socio-political affiliation–the “archaeological site” within which the original inhabitants of the region resided, and sometimes the more specific context (such as a structure or feature) with additional cultural connotations. Zooarchaeological and paleontological specimens are additionally recorded in terms of the vertical location that defines their relative association with different geological or cultural strata. However, while paleontological specimens are temporally defined in terms of broad geologic strata, archaeological specimens are temporally defined in both chronometric (absolute date) and culturally relative dates that are linked to the history of site occupancy. These terms are important designators for zooarchaeological datasets, but their terminology and associated information are best captured through the archaeological data repositories and publishers to which ZooArchNet links with shared persistent identifiers (to be discussed below).

Therefore, ZooArchNet is designed to accommodate the designation of datasets from various conceptions of site and collection contexts and to ensure effective presentation of a range of associated metadata at various levels (e.g., collection metadata, project or site metadata, or temporal metatdata). For example, a zooarchaeological dataset may include all specimen records from a given archaeological site or just a portion of a site (e.g., one of ten features or strata), or all specimen records from a geographically regional and/or temporally defined “collection” (e.g., zooarchaeological records from Florida archaeological sites or Woodland Period sites). Consistent with all other records, a zooarchaeological dataset may also encompass all records from an institutional data provider and umbrella collection (e.g., Environmental Archaeology Collection at the Florida Museum) or from an individual contributor. Special needs for representing heterogeneity must be addressed both in metadata descriptions and standardizing datasets, which are the two key steps in publication discussed in detail next.

#### Metadata and zooarchaeological datasets

Ideally, zooarchaeological dataset-level metadata published through ZooArchNet includes, in addition to the traditional biodiversity-related metadata normally published by VertNet, descriptions of the site and/or context-level excavation and sampling process, including reported units, values, and observations, as well as details specific to the specimen-level archaeological provenience including context interpretation, chronology, specific methods of excavation or sampling, and the methods of zooarchaeological analysis. Accessibility to metadata is crucial to the successful use of zooarchaeological sample data across disciplines (e.g., [33,38]). However, similar to determining the scope of a dataset, the range of metadata information and level of detail available is highly variable between original data sources, and many published zooarchaeological datasets do not include this full suite of data and associated information (e.g., specific laboratory protocols and methods of analysis [36,39]). Therefore, how it is reported in ZooArchNet is subject to the discretion of the data provider. This is especially the case when publishing legacy data or data from long-term curated faunal assemblages no longer directly attached to the original project excavator or zooarchaeological analyst.

As part of the publication process of VertNet, dataset level metadata for ZooArchNet is reported through the GBIF IPT metadata resources categories available for all published collections (e.g., http://ipt.vertnet.org:8080/ipt/). For the most part, dataset level metadata for zooarchaeological materials are reported as they are for any other biodiversity dataset. However, ZooArchNet incorporates a few critical variations to ensure effective and ethical capture and use of important information. The most important of these is an ethical issue. As is the case with publishing data regarding archaeological remains in general, when reporting the geographic location of origin (e.g., the archaeological site), it is vitally important that possible ethical considerations pertaining to location disclosure be considered. Just as with sensitive biological taxonomic groups, culturally valuable heritage site locations must be protected from illegal looting or unintentional damage by visitors. However, additional concerns by some cultural groups about disclosure of sacred site locations must also be taken into account. Open Context hosts and co-developed the Digital Index of North American Archaeology, a gazetteer of archaeological sites based on data aggregated from US State and Federal government sources [40]. Together with stakeholders in the Tribal and the State Historic Preservation Office system, Open Context and the Digital Index of North American Archaeology (DINAA) have developed a set of mutually accepted guidelines appropriate for publication of site locations that ZooArchNet follows (see http://ux.opencontext.org/archaeology-site-data/dinaa-sensitive-data-security-measures-and-shpo-collaboration/). As a general rule, in ZooArchNet, geographic coverage is buffered by an area much larger than the actual site (typically a 20km boundary) and this generalization is also reported at the record level in Darwin Core fields dataGeneralization, informationWithheld, and coordinateUncertaintyInMeters.

Reporting methods of zooarchaeological specimen recovery and analysis includes both the archaeological methods used to recover the physical specimens and the techniques used to process, identify, and analyze the specimens. The IPT metadata categories include Sampling Methods and Additional Metadata sections that provide a location to capture this essential content. Each section provides great flexibility in terms of the type of information reported and breadth of detail. The ZooArchNet workflow, when reporting methods of analysis, is to enter the broader archaeological methods of excavation, sampling, and recovery techniques under Sampling Methods and the zooarchaeological methods of analysis under Additional Metadata.

A fully worked example of dataset level metadata for a set of zooarchaeological specimens recovered from Parnell Mound, a Suwannee Valley archaeological culture site near White Springs, Florida, can be viewed at http://ipt.vertnet.org:8080/ipt/resource?r=flarch_zooarch_parnell_feature1. These materials all came from a single feature within the site, which is a pit approximately 2.5 m by 3 m in diameter extending half a meter below the former ground surface. This is the only such feature at the site. The dataset level metadata thus provides context about both the main Parnell site and the feature.

It is critical to note that some metadata is not reported at the dataset level but at the record level, and that occasionally the same metadata is reported at both levels. This is because searches in portals with aggregated data often return records from hundreds or thousands of collections, which would make assembly of individual dataset metadata extremely cumbersome. Instead, content such as the data license is often reported both at the dataset and record levels. In the case of information such as provenience or chronology, these may be assembled at a broader spatial, cultural or taxonomic unit, but are also reported at the specimen level as well. This has implications for making key provenience and chronology metadata available for search and discovery. Sometimes these attributes are indexed at the specimen level, and sometimes these attributes are indexed only at the more general dataset level.

#### Cross-walking and migrating zooarchaeological datasets into the biodiversity data network

Converting zooarchaeological specimen records stored in local databases and spreadsheets into a common format is a multi-step process. At heart of the ZooArchNet this process is aligning as much of the record as possible to Darwin Core terms, as with any biological dataset mobilization process. Through direct collaborative work between the data providers and VertNet/ZooArchNet data publishers, the first step in this data management process is the reduction, or “cleaning”, of complicated datasets with layered aggregations to simplified specimen record flat files. This cleaning step is particularly important because reporting in zooarchaelogical datasets can often include aggregations above the record level, but still reported with each record. A direct example from the exemplar datasets used here is that of specimen weights. In some FM zooarchaeological datasets, these are reported for all elements per taxon, spread across multiple specimen records or rows within a given provenience (see S1 Table). Likewise, there may also be noted relationships between elements that form records that can span proveniences, such as portions of elements that cross-mend across archaeological provenience. In all cases, it is critical to properly describe element attributes such as weight, or relationships between records, in such a way that those can be reported correctly when placed in Darwin Core semantics. This step precedes any crosswalking to Darwin Core terms.

The next critical step is the “cross-walking” or mapping/alignment of fields in the original datasets with the field structure of the Darwin Core. As specified in the beginning of this discussion, the ZooArchNet goal is to mobilize through VertNet and the biodiversity networks, all specimen-related biological data and directly associated locality and temporal data to contextualize the biological data in space and time. Through our work with multiple datasets, we identified three core concepts in zooarchaeological specimen data where Darwin Core lacked appropriate terminology for data reporting: provenience, chronology, and direct information about the specimen (rather than the organism implied by the specimen) such as material condition and skeletal element.

We also identified a significant misalignment between the DwC field, dwc:basisofRecord, and the ability to explicitly report or search for an “archaeological” specimen. This misalignment is important because it is used for indexing the final published data. Zooarchaeological records are a novel addition to the vast body of biodiversity data that have been mobilized to date. Because their nature extends the concept of biodiversity occurrences into the hitherto unrepresented archaeological context, there is a need to be able to identify these records based on that characteristic. In Darwin Core, the usual way to distinguish the nature of the biodiversity record is through the value given in a record for the term basisOfRecord. To date, the closest match for zooarchaeological records is “FossilSpecimen” (http://rs.tdwg.org/dwc/terms/FossilSpecimen), and this is what is currently being used to characterize these records. Effort is underway to submit a proposal to the Darwin Core Maintenance Group for a new value for controlled vocabulary of the basisOfRecord term, “ArchaeologicalSpecimen”, to highlight the distinct nature of these records in biodiversity applications. In the interim, the current VertNet portal now has a special flag, “isarch”, that can be used for searching via the portal (or API) to discover records of archaeological origin along with the wide variety of other attributes supported in searches. Once the new basisOfRecord value is ratified and incorporated into the standard and into the published data sets, this flag will no longer be necessary.

The specifics of how we created element, provenience and chronology data are also critical to describe in detail here, since these are essential to the correct interpretation and use of the records. ZooArchNet places data about material condition and elements in the Darwin Core term dwc:preparations, as is currently common practice with paleontological specimens.

For reporting elements, we added a field called ‘Element URI’ to datasets prior to Darwin Core migration, which converted into a key-value pair in the Darwin Core field *preparations*. The value of ‘Element URI’ is an identifier from the Uberon ontology, a cross-species reference for anatomy [28]. This ‘Element URI’ linkage allows us to clarify what is meant by a certain anatomical term, and in the future, it could facilitate an ontologized search capability for elements (see Table 2). Such a development would be highly significant within archaeology, where zooarchaeological element data is key to studying enduring topics such as process of animal domestication. In fact, the significant utility of this approach has been successfully demonstrated in the Central and Western Anatolian Neolithic Working Group, which used Uberon terms to link more than 200,000 anatomical elements in zooarchaeological datasets from 17 sites in Turkey [41], contributing to our understanding of the westward spread of domestic animals during the Neolithic [42].

**Table 2:** Example of the data cleaning process.

Taxon	Verbatim Element^a^	Element	Element URI
Mugilidae	Vertebrae	Vertebra	UBERON:0002414

^a^An example from North Midden, a zooarchaeological site from Florida, which illustrates how we transform the ‘Verbatim Element’ value from the data contributors into a “cleaned” ‘Element’ value, which can then be matched using an automated process in R to its appropriate Uberon identifier (shown in the field ‘Element URI’).

There are no Darwin Core terms directly related to provenience or chronology although currently such information can be captured in dwc:dynamicProperties as structured content in a standard syntax. For these critical data, we have created standardized content and show an example of key fields from an already published specimen in Table 3 with a more detailed explanation of the meaning there. We note two key aspects of standardizing reporting. The first is that the use of JSON for the string in the Darwin Core fields such as preparations and dynamicProperties that allows us to present nested and relatively dense information that can be reformatted to view in many JSON viewers. VertNet has also developed tools to extract and index valuable additional attribute data from these fields even though they typically lack internal standardization [43]. Second, there is a necessary mix of standardized and non-standardized reporting. All provenience information in dwc:dynamicProperties is prefaced with the term “provenience”. However, the nested content within provenience is not standardized, since there is no community controlled vocabulary for how that is reported. By contrast, we have developed a controlled vocabulary for chronometry reporting which uses widely accepted shared terminology across archaeology and paleontology. We provide both a standard term, "ChronometricDates", and also standardize all the nested terms to facilitate search. Full discussion on chronometry standardization is the subject of a separate contribution (Brenskelle et al., in prep).

**Table 3:** Example fields extracted from a ZooArchNet published specimen.

Field	Value
**ScientificName:**	Odocoileus virginianus
**DynamicProperties^a^:**	{"Site Number":"8CO326", "Provenience": {"Test Unit":"TU 17", "Level":"H", "Stratum":"V"}, "Provenience Notes":"1x1 m unit in the center of Feature 1. NE corner local grid coordinates: 1003.42m E, 1019.19m N", "Level and StratumDescription":"10YR2/1 black greasy sand and abundant charcoal; articulated deer vertebrae and several other complete bone specimens", "Sum weight in grams of all elements in catalog number for taxon":" 182.68", "ChronometricDates": [ {"maximumChronometricAge":"1152", maximumChronometricAgeReferenceSystem:"AD", "maximumChronometricAgeReferenceSystem":"AD", "minimumChronometricAge":"1260", "minimumChronometricAgeReferenceSystem":"AD", "chronometricAgeUncertaintyInYears":"30", "materialDated":"Charcoal found in Stratum V", "chronometricDateProtocol":"Association with AMS dated portion of Stratum V", "chronometricDateReferences": "Wallis, N.J. and M.E. Blessing. 2015. Ritualized Deposition and Feasting Pits: Bundling of animals in Mississippi Period Florida. Cambridge Archaeological Journal 25(1):79–98.; Wallis, N.J. and M.E. Blessing. 2015. Big Feasts and Small Scale Foragers: Pit features as feast events in the American southeast. Journal of Anthropological Archaeology 39:1–18.", "chronometricDateRemarks":"Beta Analytic number—323913"}] }
**EventDate:**	1160/1260
**LocationID:**	https://opencontext.org/subjects/e54377f7-4452-4315-b676-40679b10c4d9

Example fields extracted from a published zooarchaeological specimen http://portal.vertnet.org/o/flarch/parnell-feature1?id=00a28159-a25c-4ab1-ae85-f4c0f1c4db64 from the “UF Florida Archaeology Parnell Site (8CO326), Feature 1 Zooarchaeological Data” dataset (http://ipt.vertnet.org:8080/ipt/resource?r=flarch_zooarch_parnell_feature1)) showing format of key fields for archaeological data. The fields dwc:preparations and dwc:dynamicProperties contain key-value pairs (e.g., the key is “Element” and the value is “Radius” for the first pair in dwc:preparations). The content has been formatted for readability but is identical to what is in the record. Note that the dwc:preparations field contains a link to a term in UBERON, which when resolved (http://purl.obolibrary.org/obo/UBERON_0001423) provides a definition of the class “radius bone”.

^a^The field dwc:dynamicProperties contains detailed information on provenience, weight and chronometric dates. We note, in particular, that weight is defined as “sum weight in grams of all elements in catalog number for taxon". In this case, there are 15 other occurrence records that bear that same catalog number, and it is the weight of all of the *Odocoileus virginianus* for all 15 of those records that totals the measured 182.68 grams. The fields dwc:eventDate and dwc:locationID contain single values, and in the case of the date it reflects when the Occurrence was in its context. The dwc:locationID field contains a URL which links this record to OpenContext, as discussed in more detail in the text.

#### LOD application and linking to Open Context as an exemplar archaeological open data publisher

The ZooArchNet implementation relies on the foundations provided by the biodiversity and archaeological data communities, and in both of these, Linked Open Data is still nascent. VertNet has limited capacity for providing true Linked Open Data capacity as it currently stands, but we note that even with this limitation, attempts are being made to support critical steps towards LOD ideals. The current logical field to support linking digital records is the dc:references field, and this field often contains a self-reference to the resolvable VertNet record. Unfortunately, this record cannot be guaranteed persistent and only resolves HTML. A longer term solution is resolvable occurrence identifiers in the DwC:occurrenceID field, which has been the subject of much debate and contention in the biodiversty informatics literature (summarized in [44]). Neither VertNet nor any biodiversity data infrastructure has yet been able to provide machine-readable metadata from occurrence identifiers. While this is not an ideal state of affairs, minted persistent and globally unique identifiers for terms such as DwC:locationID, and as discussed more below, can serve as a means to develop the first steps towards a more realized Linked Open Data future for zooarchaeological data resources.

On the archaeological side, the DINAA gazetteer that is part of Open Context already publishes data on a large compendium of site records based on data aggregated from US State and Federal sources [40,45,46], each of which has a unique URI that can be resolved in variety of formats (HTML, GeoJSON, JSON-LD, RDF-XML, N3, etc.) to facilitate Linked Data applications. Open Context creates persistent identifiers for sites, site contexts, and each individual specimen, among many other data points. By using an existing identifier for a site in Open Context, when such already exists, as the dwc:locationID published through VertNet/ZooArchNet, biodiversity occurrence records in Darwin Core can directly reference the information-rich archaeological site and context records in Open Context or any other archaeological publisher that references that same location identifier. The process of assigning *locationID*s in the ZooArchNet process consisted first of a search for the relevant site in Open Context (for example, Parnell Feature 1,https://opencontext.org/subjects/e54377f7-4452-4315-b676-40679b10c4d9). If the correct site was found, the URL identifier for that site was used as the dwc:locationID for the biodiversity record. If the site was not found in Open Context, a new site URL identifier was created in Open Context, and then the same process of using that site identifier for the dwc:locationID was employed. At times the site existed in Open Context, but the provenience of the biodiversity records were from a sub-site (for example, the Parnell Feature 1, a sub-site of the Parnell site [https://opencontext.org/subjects/B5F813A9-4273-4725-8885-80176BE5668E#tab_obs-3]). Open Context permits hierarchical levels of context, facilitating creation of a sub-site in Open Context and thus the dwc:locationID in the biodiversity record in VertNet/ZooArchNet can link directly to that specific context in Open Context. If VertNet/ZooArchNet creates a new dataset that does not yet have a site identifier available on Open Context or any other repository, the identifier can be minted by whomever is best suited to manage site level metadata, which is likely to be Open Context or allied repositories.

## Discussion and conclusion

ZooArchNet provides a novel workflow and platform for publishing zooarchaeological records to global distributed biodiversity data networks with intact integrated and linked archaeological information. Zooarchaeological data is sometimes already published in paleo- and biodiversity repositories, but in most cases, these records are disconnected from the cultural and methodological content needed for proper interpretation. Conversely, when biological data is published through archaeological platforms, the rich information about biodiversity samples are often also not fully available. However, current practice of sundering zooarchaeological records into “biodiversity records” or “archaeological records” is not an inevitable consequence of publishing records in a particular format or for a particular user-base. Rather, it has been due to lack of collaborative work to tackle the very real challenges of redefining the use of existing but very different standards and infrastructure to report needed data, and the challenge of connecting methods and research questions across disciplinary divides. Underappreciated is the fact that standards and data repositories can be a means to *close* rather than widen those divides, by recognizing common languages, creating ways to share those languages and to develop alignments between different languages, and fostering new ways to re-use data to meet common and discipline specific goals.

The timing on developing such approaches to link across data resources is particularly critical because both archaeological and biodiversity science have become more integrative, and this work requires assembling data across disciplinary divides and variable sources of data [47]. Therefore, conceptually and in practice, we have designed ZooArchNet not to be “yet another portal or data repository” but rather to serve as a *bridge* between publishing mechanisms already established by both the natural history and archaeological research communities. This is a critical design goal that facilitates long-term interoperability of data, emphasizing the importance and crucial role of persistent identifiers in facilitating data discovery across disciplines. In particular, we note the value of sharing identifiers that define both archaeological sites and material samples. While the concept of an archaeological site can be complex, composed of hierarchically organized provenience designations across various archaeological contexts, the use of unique site identifiers and specimen occurrenceIDs, as well as explicitly associated vocabulary standards, creates the ability to link and search for specimen data from potentially multiple scales of archaeological resolution, including within and between proveniences at a given site. This ability creates essential pathways to discover and maintain the contextual integrity of zooarchaeological specimen records across paleontological, neontological and archaeological repositories.

In addition to the advancement of open data bio- and archaeoinformatics, ZooArchNet is meant to be catalytic in meeting existing and future research needs focused on examinations of human impact on, and responses to, animal biodiversity over long time-scales [48,49,50]. To that end, while we expect focused data-oriented research projects in the future, simply exposing the wealth of physical zooarchaeological material and cultural data available across a diversity of data repositories has real benefit for the greater biodiversity and archaeological research communities. This is especially true given not only the creative approaches now possible to extract new information from zooarchaeological specimens themselves (e.g., ancient DNA, ZooMs), but also in terms of the increasingly innovative approaches being developed to model climate change and human responses across time, space, and people utilizing interdisciplinary datasets and research design [51,52,53,54,55]. Zooarchaeological data are a critical component in such research endeavors, and particularly for the creation of historically derived “baseline” data used to help predict the possible ranges of and relationships between environmental, animal, and human responses and adaptations to climate change worldwide. ZooArchNet thus enables the efficient dissemination, exploration, and use of in demand primary zooarchaeological specimen records that are often difficult to discover or access.

Our efforts at publishing zooarchaeological datasets, and building new infrastructure to support that publishing, are meant to be proof of concept and focused on Florida collections in an effort to produce immediately usable regional data. Florida is exemplary of coastal landscapes worldwide with deep histories of complex climate- and anthropogenic-induced biodiversity changes. Currently across the state, but particularly among coastal habitats, people and animals are contending with rising sea levels and temperatures as well as increasingly variable extreme weather patterns, all of which directly impact terrestrial and aquatic environments and biota, including marine, estuarine, riverine, and freshwater coastlines, surrounding terrestrial landscapes, and associated animal biodiversity [56,57,58,59,60]. Furthermore, there are significant cultural, social, technological, and economic consequences of such environmental changes, particularly for coastal populations and industries tied to environmental and animal biodiversity (e.g., industrial fisheries, recreational fisheries, aquatic farming, ecotourism, property value and development) [61,62,63]. As is the case in other coastal locales, current approaches to identifying, combatting, and/or mitigating changes in Florida’s biodiversity include the production of historical perspectives of past taxonomic diversity and climate conditions [64,65,66], both of which are represented in zooarchaeological specimen records [67,68,69,70].

Despite our regional focus and recognizing that our efforts have so far been proof of concept, the aspiration of ZooArchNet is to provide a platform for broadly mobilizing zooarchaeological data (see zooarchnet.org). As explained above, we are focused on the development of a dedicated ZooArchNet portal, while continuing to publish specimen records and collections through VertNet. The use and development of LOD applications continues to be a focus as well, namely the use of persistent identifiers. Moving forward, we are also open to pursuing the integration of other media records (e.g., photographs) relevant to published specimen records. For example, VertNet currently supports references (e.g., links) to and metadata for media, but the media have to be accessible online independently of VertNet (and ZooArchNet). This is achieved through dwc:associatedMedia in Darwin Core and through the Audubon Media Description extension to Darwin Core (https://tools.gbif.org/dwca-validator/extension.do?id=http://rs.tdwg.org/ac/terms/Multimedia).

In closing, the three keys aspects of our publishing ethos are: 1) Our goal is to create linked open data from the outset and as such ZooArchNet is not a siloed infrastructure, but rather intends to connect explicitly to other open data repositories such as Open Context and DAACS. At the same time, by publishing in ZooArchNet (as subset of VertNet), data are also made available to the global research community via publishing systems that assure datasets are also registered and made available in VertNet and GBIF. Further efforts are still needed, but soon underway, to better connect to paleontological resources such as those found in Neotoma [71]. 2) Besides a reliance on site identifiers and specimen identifiers, which form the core of describing the context for material samples, ZooArchNet is flexible about what gets published, from individual units within an excavation to the largest collections housed in museums. 3) The publishing flexibility reflects a strong belief among the ZooArchNet informaticians and researchers in the necessity of “meeting the community where it is” with regards to supporting data publishing. This means that all data publishing and continuing efforts to improve that process are shared efforts that we argue offer strong and tangible good for the future of the connected disciplines around biodiversity and human impact on our planet.

## Supporting information

S1 Table The full spreadsheet from the Parnell site, an archaeological site in Florida. This shows the cleaned dataset before the Darwin Core cross-walking is complete. Note the 'Verbatim' and 'Clean' Taxon and Element fields, which shows how these fields are edited slightly in order to accommodate the UBERON mappings for element and the VertNet propagation for scientific name fields.
(XLSX)
